# The Minnesota Haptic Function Test

**DOI:** 10.3389/fpsyg.2019.00818

**Published:** 2019-04-17

**Authors:** Jessica Holst-Wolf, Yu-Ting Tseng, Jürgen Konczak

**Affiliations:** ^1^ Human Sensorimotor Control Laboratory, School of Kinesiology, University of Minnesota, Minneapolis, MN, United States; ^2^ Department of Physical Education, National Tsing Hua University, Hsinchu, Taiwan; ^3^ Research Center for Education and Mind Sciences, National Tsing Hua University, Hsinchu, Taiwan

**Keywords:** active touch, human, perception, proprioception, somatosensation, tactile

## Abstract

Haptic loss severely compromises the fine motor control of many daily manual tasks. Today, no widely accepted assessment protocols of haptic function are in clinical use. This is primarily due to the scarcity of fast, objective measures capable of characterizing mild to severe forms of haptic dysfunction with appropriate resolution. This study introduces a novel curvature-perception assessment system called the Minnesota Haptic Function Test™ that seeks to overcome the shortcomings of current clinical assessments.

**Aims:** The purpose of this study was threefold: (1) apply the test to a sample of young healthy adults to establish test-specific adult norms for haptic sensitivity and acuity; (2) establish the reliability of this instrument; (3) demonstrate clinical efficacy in a limited sample of cancer survivors who may exhibit haptic dysfunction due to chemotherapy-induced peripheral neuropathy.

**Method:** Participants manually explored two curved surfaces successively and made verbal judgments about their curvature. A Bayesian-based adaptive algorithm selected presented stimulus pairs based on a subject’s previous responses, which ensured fast convergence toward a threshold. Haptic sensitivity was assessed by obtaining detection thresholds in 26 adults (19–34 years). Haptic acuity was assessed by obtaining just-noticeable-difference thresholds in a second sample of 28 adults (19–25 years). Nine cancer survivors (18–25 years) with suspected peripheral neuropathy completed the acuity assessment. Test-retest reliability of the algorithm was calculated.

**Results:** First, the test yielded values that are consistent with those reported in the literature. Mean detection threshold for curvature of the healthy adults was 0.782 (SD ± 0.320 m^−1^). The corresponding mean discrimination threshold was 1.030 (SD ± 0.462 m^−1^). Second, test-retest reliability of the algorithm was assessed in a simulation, yielding an average correlation between repeated simulated thresholds of *r* = 0.93. Third, the test documented that 86% of the cancer survivors had acuity thresholds above the 75th percentile of the normative cohort, and 29% had thresholds above the normal range, indicating that the instrument can detect and differentiate between unaffected perception, and mild or more severe forms of haptic loss.

**Conclusion:** We here provide evidence that this new method to assess haptic perception of curvature is valid, reliable, and clinically practicable.

## Introduction

Haptics, also called “active touch,” refers to one’s ability to extract object features such as shape, orientation, hardness or softness, and texture by moving the hands or other body surfaces around an object (Gibson, 1966). Haptic perception is multimodal and depends primarily on two somatosensory modalities, proprioception and touch. Proprioception is the perception of body and limb orientation and movement. It is based on signals from mechanoreceptors in skeletal muscle fibers, tendons, skin, and joints. The sense of touch is informed by signals from four types of mechanoreceptors embedded in the human glabrous skin that encode object features such as shape, motion of objects in contact with the skin, skin stretch, low- or high-frequency vibration, and texture. Characteristic manual exploratory procedures and associated processes of sensory integration of proprioceptive and tactile information give rise to haptic perception (Lederman and Klatzky, 1993, 2004). Thus, haptics has also been described as *active* or *dynamic touch* to perceive object characteristics.

Many tasks of daily living involve object manipulation and require functional haptic perception (Klatzky et al., 1985; Kalisch et al., 2012). Despite the recognized importance of haptics for object manipulation, there is no universally accepted measure of haptic function given that there are multiple forms of haptic perception such as shape or texture. Moreover, haptic function is difficult to measure quickly and with appropriate resolution in a clinical setting. Given the lack of a widely accepted haptic function assessment tool, there is no definitive characterization of haptic ability across age or for clinical conditions that are known to affect somatosensory function.

Numerous diseases of the peripheral or central nervous system such as diabetes (Travieso and Lederman, 2007), Parkinson’s disease (Konczak et al., 2012), dystonia (Putzki et al., 2006), and stroke (Meyer et al., 2014) are known to degrade haptic perception. In pediatrics, conditions such as developmental coordination disorder (DCD) (Wang et al., 2009; Li et al., 2015; Tseng et al., 2018), cerebral palsy (Goble et al., 2009), and chemotherapy-induced peripheral neuropathy (CIPN) (Moore and Groninger, 2013; Smith et al., 2015) are associated with proprioceptive and haptic deficits that impair motor behavior and motor development. Consequences of haptic impairment can be significant and detrimental. DCD is associated with negative impacts on psychological, social, and physical function compared with age-matched children (Zwicker et al., 2013). Surveys on long-term survivors of pediatric cancers found that adult survivors are more likely than their non-treated siblings to report limitations that affect self-care, performance of routine activities, and the ability to attend work or school (Ness et al., 2005). As haptic impairments can have significant effects on quality of life, the ability to identify and monitor changes in haptic function in these populations is critical for comprehensive, long-term care.

The ideal haptic test will yield an objective high-resolution measure of function that correlates with functional upper limb impairment, is easy to administer, and can be completed quickly in a clinical setting. Several groups have created assessments of haptic function including tests to measure aspects such as texture perception, object recognition, curvature detection or discrimination, and two-point discrimination but none have become a universal standard (Gordon and Morison, 1982; Lamb, 1983; Miyaoka et al., 1999; Ballesteros et al., 2005; Soechting and Poizner, 2005; Lederman and Klatzky, 2009) There are numerous reasons why these tests have not been widely adopted such as the use of expensive or large equipment, time-consuming test procedure, or the lack of sensitivity. In addition, clinicians desire an instrument that links somatosensory impairment to motor dysfunction. Tactile assessments such as texture perception have not correlated with upper limb motor function. In that respect, assessments of tactile function such as texture perception are not well suited because it does not correlate well with motor function (Auld et al., 2012).

To address clinical needs, the Minnesota Haptic Function Test™ was designed to characterize the shape-form haptic function using a curvature perception task. This sub-modality of haptic perception was selected for several reasons. First, the shape-form aspect of haptic function requires multimodal processing of both tactile and proprioceptive information. Shape-form information plays a direct role in object manipulation and tasks of daily living, making this aspect of haptic function critical for upper limb motor function. Second, curvature detection and discrimination tasks easily accommodate new psychophysical threshold searching methods (Prins, 2013) that generate high-resolution thresholds of haptic function in a short period of time. The aims of this study were twofold: first, to present the methodology of the Minnesota Haptic Function Test™; second, to establish the validity, resolution, and test-retest reliability and create a normative dataset of typical haptic function in young adults. Our assessment provides a measure of haptic sensitivity, a detection threshold, and a measure of haptic acuity, a discrimination threshold. Here, sensitivity is defined as the smallest convex stimulus an individual can reliably perceive as curved compared with a flat surface (Ehrenstein and Ehrenstein, 1999; Konczak et al., 2012). Acuity is defined as the smallest difference between two perceptibly curved surfaces an individual can reliably perceive.

### Description of Instrument

**Figure 1:**
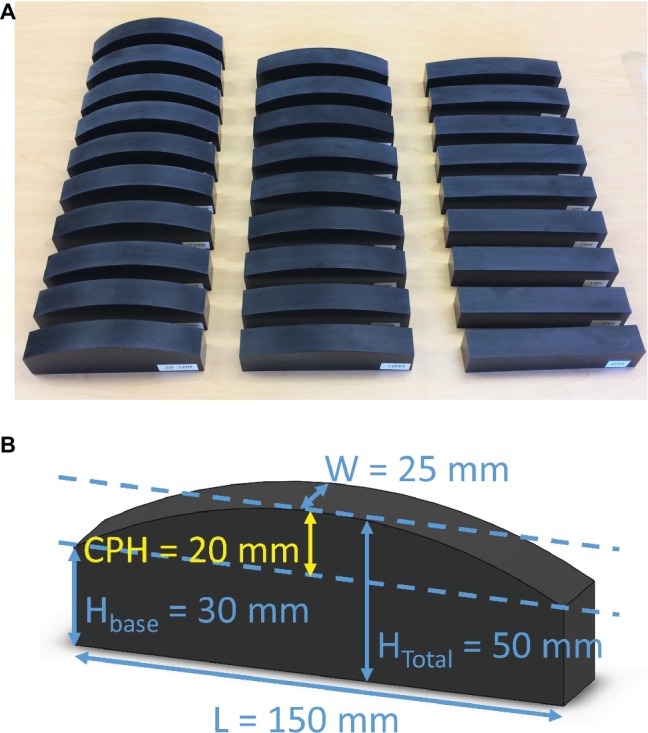
**(A)** The Minnesota Haptic Function Test™ curvature block system. The system consists of 28 blocks with center-point-heights ranging from 0 mm (flat) to 34 mm. **(B)** Dimensions of a sample block from the system with 20-mm center-point-height (label CPH). Each block has the same base dimensions: W = 25 mm, H_base_ = 30 mm, and L = 150 mm.

The assessment system consists of 28 high-precision, custom-made plastic blocks. The bases of the blocks have identical width, length, and height (25 mm × 150 mm × 30 mm). Each block has a defined curvature with a defined curvature center-point-height (CPH) ranging from flat (0 mm) to maximally curved (34 mm, see Figure 1). The curved surfaces of these blocks are circular arcs, meaning they represent a part of a circle’s circumference (i.e., each center-point-height corresponds to a circle with a different radius). The standard measure of curvature is the inverse of this radius and the unit is m^−1^. For the sake of simplicity and consistency with previous reports (Gordon and Morison, 1982), we here use CPH to differentiate between blocks. The CPH tolerance is < ±0.1 mm for all whole-millimeter center-point-height blocks and < ±0.05 mm for the 0.5-mm center-point-height blocks. Table 1 provides a complete description of the block system with center-point-heights, tolerances, and the associated curvature values. When not in use, the block system is stored in a rolling case, making it transportable and easy to store. The assessment requires only a small footprint and can be performed in small spaces. The only requirements are a flat surface to place the blocks and a height-adjustable chair for the participant. In addition, a laptop computer is needed. The examiner records the binary responses using Matlab Technical Programming Language software. The Matlab software determines the order of the next stimuli pair and the size of the comparison stimulus (see the following section for further details).

**Table 1:** The Minnesota Haptic Function Test™ block dimensions. All blocks have a base width of 25 mm and length of 150 mm.

Height (mm)	Center-point-height (CPH) (mm)	CPH tolerance (<± mm)	Curvature (m^−1^)
30.0	0.0	0.10	9.1954
30.5	0.5	0.05	9.3055
31.0	1.0	0.10	9.4139
32.0	2.0	0.10	9.6255
33.0	3.0	0.10	9.8302
34.0	4.0	0.10	10.0280
35.0	5.0	0.10	10.2190
36.0	6.0	0.10	10.4031
38.0	8.0	0.10	10.7512
40.0	10.0	0.10	11.0727
42.0	12.0	0.10	11.3683
44.0	14.0	0.10	11.6387
46.0	16.0	0.10	11.8848
47.0	17.0	0.10	11.9990
48.0	18.0	0.10	12.1075
49.0	19.0	0.10	12.2103
49.5	19.5	0.05	12.2597
50.0	20.0	0.10	12.3077
50.5	20.5	0.05	12.3544
51.0	21.0	0.10	12.3997
52.0	22.0	0.10	12.4865
53.0	23.0	0.10	12.5682
54.0	24.0	0.10	12.6449
56.0	26.0	0.10	12.7839
58.0	28.0	0.10	12.9047
60.0	30.0	0.10	13.0081
62.0	32.0	0.10	13.0954
64.0	34.0	0.10	13.1674

### Assessment Procedure and Measures

**Figure 2:**
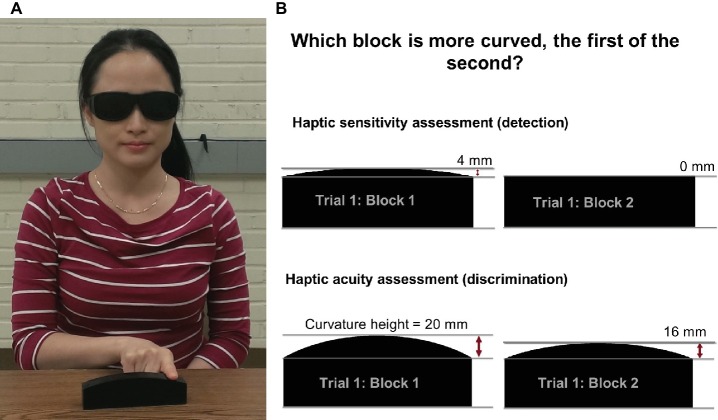
**(A)** An individual manually exploring a haptic system block using lateral movements of the index finger while wearing vision-occluding glasses. This individual provided written informed consent for the publication of his/her image. **(B)** The procedure for the haptic sensitivity and acuity assessments: two exemplary blocks presented to a participant in a single trial for the sensitivity and acuity assessments.

Participants use the index finger of the dominant hand to manually explore the surface of two curved blocks presented consecutively (see Figure 2A). The manual exploration consists of up to four lateral movements of the index/finger hand (side-to-side) with the finger in contact with the surface of the block. In each trial, two blocks are presented, the reference block and a comparison block. For the assessment of sensitivity (i.e., detection threshold), reference block is always flat (CPH = 0 mm) and the comparison block has always a convex curvature with center-point-height ≥ 0.5 mm (see Figure 2B). The reference and comparison blocks are presented in pseudorandom order with the reference block presented first for half of the trials. After completing the exploration of two blocks, the participant provides a verbal response indicating which block is more curved, the first or the second. The response is coded by the experimenter for correctness and entered into the computer. The software implements the Psi marginal adaptive algorithm and selects the stimulus intensity for each subsequent trial based on the response correctness and the previous stimuli intensities (Prins, 2013). This adaptive algorithm assures fast, near monotonic convergence toward the true threshold. After 20 trials, the software fits a logistic Weibull function to the response-stimulus intensity data and generates a haptic just-noticeable-difference (JND) detection threshold, thus, obtaining an objective measure of haptic sensitivity. The algorithm also calculates an estimate of the variability of the participant’s responses represented as the slope of the logistic Weibull threshold function. The haptic acuity assessment follows the same method as described above, but here the reference block has a 20-mm CPH. The comparison block can be smaller or larger than the reference block, or the experimenter selects an ascending or descending method, i.e., where the comparison block has a CPH that is always larger or smaller than the reference block.

During assessment, participants wear vision-occluding glasses to block any visual size cues. The blocks are placed in a consistent position near the edge of the table in order to avoid that the participant simultaneously touches the table while exploring a block and thus receiving proprioceptive size cues based on difference in wrist or finger position. The participant’s non-dominant hand rests on the lap or on the table. The participant’s chair height is adjusted in such a way that the person’s elbow is in a comfortable position at approximately 90° of flexion.

### Resolution of the Instrument

The smallest curvature block difference is 0.5 mm of center-point-height. The manufacturing tolerance of these blocks was established to be <0.1 mm. The actual measured differences was +0.03 mm for the 49.5-mm center-point-height block and +0.07 mm for the 20.5-mm center-point-height block. Even though the Psi marginal algorithm can calculate thresholds past the tenths decimal place, the recommended resolution of the thresholds is 0.1 mm, the resolution of the block system. For the calculation of the slope of the logistic fit, rounding to the nearest one-hundredth is reasonable.

## Materials and Methods

### Participants

Haptic sensitivity and acuity thresholds were measured in *N* = 26 adults (M/F: 8/18, age range: 19–34 years, mean age: 22 years, handedness R/L: 26/0) and *N* = 28 adults (M/F: 12/15, age: 19–25 years, mean age: 20 years, handedness R/L: 28/0) respectively. Four additional adults completed the acuity assessment five times to establish the assessment test-retest reliability (M/F: 2/2, age range: 23–27 years, mean age: 25 years, handedness R/L: 4/0). In addition, nine adults treated with chemotherapy for pediatric cancers with suspected peripheral neuropathy completed the acuity assessment (*N* = 9 adults, M/F: 3/6, age range: 18–25, mean age: 21 years, handedness R/L: 8/1). This provides a small heterogeneous sample of individuals at various time points during chemotherapy treatment after diagnosis with various forms of pediatric cancer such as leukemia, or lymphoma. For details of individual diagnosis and time since diagnosis, see Table 2. An additional inclusion criterion for this group was exposure to vinca alkaloids as a part of treatment. Exposure to this type of chemotherapeutic agent is known to be associated with peripheral neuropathy (Moore and Groninger, 2013). The dominant hand was used for assessment. Handedness was determined by the modified Edinburgh handedness inventory. Participants in the healthy cohort confirmed that they had no known peripheral or central nervous system condition that would affect haptic function. All individuals with suspected peripheral neuropathy were currently being treated for pediatric cancer and had been exposed to vinca alkaloids, a chemotherapeutic agent known to cause peripheral neuropathy. These individuals had no cranial cancer diagnoses. The study protocol was approved by the Institutional Review Board of the University of Minnesota. Data collection occurred at the University of Minnesota campus and at the University of Minnesota Masonic Children’s Hospital. All participants gave written informed consent in accordance with the Declaration of Helsinki.

**Table 2:** Characteristics of participants treated with chemotherapy that completed the haptic discrimination assessment.

Age (years)	Diagnosis	Time since diagnosis (months)
18	Ewing’s sarcoma	34
18	Leukemia	38
19	Hodgkin’s lymphoma	4
20	Acute lymphoblastic leukemia	14
21	Hodgkin’s lymphoma	39
21	Leukemia	20
24	Malignant neoplasm	132
24	Lymphoma, pancreatic tumor	39
25	Leukemia	47

### Procedure: Human Participant Testing

We applied the assessment procedure described in detail above. For the acuity testing, we selected comparison blocks that were always smaller than the 20-mm CPH of the reference block (≤ 19.5 mm). This assured that one obtained a stable threshold after 20 trials.

As part of establishing test-retest reliability, four healthy young adults repeated the acuity assessment five times over a 14- to 20-day period. Participants were not given any performance feedback, in order to avoid any learning. The standard deviations of each individual’s discrimination threshold were calculated to assess intra-subject reliability.

### Procedure: Computer Simulation-Based Reliability Testing

Because the Psi marginal method is a Bayesian inference-based adaptive algorithm, the threshold estimate between repeated assessments will differ slightly unless the examinee gives the exact same responses for each stimulus intensity in the exact same order each time. To assess the test-retest reliability of this algorithm with our specific testing parameters, we applied the same method of calculating the average correlation coefficient on threshold data generated by simulation. In this simulation, the Psi marginal algorithm received four sets of responses that were consistent with four different thresholds (1, 2, 3, and 4 mm). The algorithm was executed five times for each of the four thresholds. All trials equal to or above the set threshold were answered correctly. All trials below the threshold were answered correctly 40–60% of the time (similar to random guessing). The order of the correct or incorrect responses was varied based on pseudorandomization procedure. The average correlation coefficient between each pair of simulated threshold tests (T1-T2, T1-T3…T4-T5) was calculated to assess test-retest reliability (5 repetitions = 10 unique between-test comparisons and thus 10 unique correlation coefficients). This provides insight into the best-case scenario for repeatability of the threshold estimation using this algorithm with the exact parameters applied here (number of trials = 20, algorithm version = Psi marginal, estimating both threshold and slope, the available stimuli intensities, and the inclusion of a fixed lapse rate = 3 trials).

## Results

### Haptic Function of the Healthy Adult Samples

**Figure 3:**
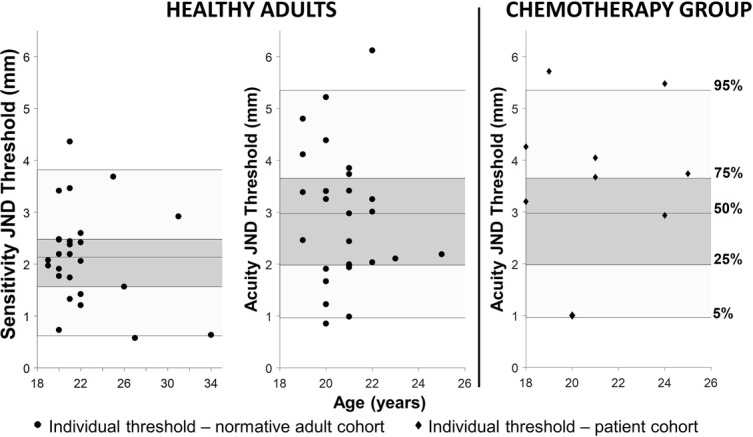
Left: the JND haptic detection and discrimination thresholds for the healthy adults. Individual thresholds are black circles. The shaded areas in all three graphs represent the quantiles generated by the healthy adult data (dark gray represents 25–75% and light gray represents 5–95%) and the center line inside the dark gray area is the 50th percentile. Right: the black diamonds in the graph on the far right are the individual thresholds from adults with suspected chemotherapy-induced peripheral neuropathy (CIPN). Note that the majority of these individuals are above the 75th percentile for the normative cohort and two are above the 95th percentile indicating haptic impairment.

All participants completed the assigned haptic function test within 10–12 min. The mean haptic detection threshold was 2.2 mm (SD ± 0.9 mm) with a mean slope of 1.06 (SD ± 0.31). The mean discrimination threshold was 2.9 mm (SD ± 1.3 mm) with a mean slope of 1.25 (SD ± 0.50). Based on these datasets, the respective 5th, 25th, 50th, 75th, and 95th percentiles for detection and discrimination thresholds in healthy adults were calculated (see Figure 3 and Table 3). No significant correlation was found between the slope values and the associated haptic thresholds (*r*_detection_ = 0.17, *r*_discrimination_ = 0.22). This indicates that the variability of a participant’s responses was not correlated with the threshold (i.e., higher thresholds were not associated with more variable responses during the assessment).

**Table 3:** Adult group haptic JND acuity and sensitivity threshold quantiles.

Quantile (%)	JND sensitivity threshold (mm)	JND acuity threshold (mm)	JND sensitivity threshold (m^−1^)	JND acuity threshold(m^−1^)
5	0.62	0.96	0.2204	0.3413
25	1.56	1.98	0.5544	0.7035
50	2.13	2.97	0.7567	1.0543
75	2.48	3.65	0.8808	1.2947
95	3.82	5.35	1.3547	1.8926

### Haptic Acuity in Adults With Suspected Peripheral Neuropathy

Six out of nine adults (67%) treated with chemotherapy for pediatric cancers exhibited acuity thresholds at or above the 75th percentile of the healthy adult cohort. Two out of the nine individuals treated with chemotherapy (29%) were above the 95th percentile of the normative cohort (see Figure 3).

### Intra-rater and Test-Retest Reliability

Four adults completed the haptic acuity assessment five times on five different days over the course of approximately 14–20 days. The mean SD across all repeated tests for the four participants was computed as 0.98 mm (SD_max_ ± 1.4 mm; SD_min_ ± 0.60 mm). These values reflect the human variability in responses that can be expected between repeated tests when tested using a block with a CPH = 20 mm as the reference.

Four adults completed the haptic acuity assessment five times on five different days over the course of approximately 14–20 days. The mean SD across all repeated tests for the four participants was computed as 0.98 mm (SD_max_ ± 1.4 mm; SD_min_ ± 0.60 mm). These values reflect the human variability in responses that can be expected between repeated tests when tested using a block with a CPH = 20 mm as the reference.

To confirm appropriate test-retest reliability of the assessment algorithm itself, the average correlation value between simulated acuity tests was calculated as *r*_mean_ = 0.93 (*r*_min_ = 0.87, *r*_max_ = 0.99), indicating that the algorithm yields high test-retest reliability.

## Discussion

### Validity of Haptic Function Measures

Currently, no standardized haptic curvature assessment is in use, but several research groups employed similar haptic curvature perception measures that can provide a standard of comparison for this assessment protocol. One report tested haptic curvature perception in 17 adults using small (20 mm length), flat, and convex glass lenses. The mean curvature detection threshold was reported to be 0.09 mm (SD ± 0.03 mm) CPH, while the mean curvature discrimination threshold was 0.12 mm (SD ± 0.04 mm) using a 0.14-mm CPH reference lens (Gordon and Morison, 1982). These thresholds are roughly an order of magnitude smaller than the mean haptic sensitivity and acuity thresholds reported here (sensitivity: 2.2 mm, acuity: 2.9 mm). However, the length of the curve was roughly one-tenth that of the curved blocks used in our protocol.

Another study reported curvature detection thresholds in the range of 1–3 mm in three participants for Gaussian curves 40–100 mm in length (Louw et al., 2000). While not round, these curves have a similar profile and scale to the blocks used in this assessment. Moreover, the magnitude of the curvature detection thresholds reported by Louw et al. (2000) is in the same range as the thresholds obtained through our assessment procedure.

Sciutti et al. (2010) used a robotic manipulandum that created “virtual” haptic curves that participants would actively explore *via* a manipulandum handle. They report a range of haptic curvature discrimination thresholds from 3 to 8 mm for a reference of 20-mm lateral deviation (the analog to center-point-height). These thresholds are slightly higher than our mean value (2.9 mm), but the curvature length was larger (200 vs. 150 mm in our test). We conclude that, while there is no direct comparison available for our haptic curvature system, the threshold values obtained appear to scale appropriately with available data for haptic curvature perception.

### Reliability

To assess how much inconsistency was due to the individual factors compared with the threshold searching algorithm, we conducted the test-retest reliability simulation. The average correlation value for this simulation was *r*_mean_ = 0.93, indicating the assessment algorithm is reliable for estimating haptic thresholds.

### Some Insights and Considerations When Applying the Assessment

Participants were allowed to self-select finger movement velocity and the number of times the block surface was traversed (up to a maximum of four times). Thus, we constrained the number of maximum surface scans by the finger in order to standardize the procedure. However, we did not control for the velocity of the movement, because doing so would require monitoring velocity during testing, which would necessitate a wearable sensor mounted on the finger. In our opinion, this would take away from the simplicity of the test procedure. In addition, it has been shown that movement speed does not influence haptic curvature perception (Soechting and Poizner, 2005).

At this point, the smallest testable stimulus intensity is 0.5 mm. When testing for detection thresholds, we found that one participant performed well enough that the adaptive algorithm selected a 0.25-mm stimulus difference indicating that the inclusion of a 0.25-mm center-point-height block in future sensitivity testing is necessary.

### A Rationale for Assessing Haptic Curvature Perception in Clinical Settings

There are multiple forms of haptic perception such as the perception of texture, hardness, or shape. Haptic object recognition using the hand is a form of haptic perception that is similar to the haptic shape assessment (Lederman and Klatzky, 1987, 1993). Assessments of haptic object recognition provide information about the functionality of haptic perception, but they do not easily provide measures of acuity and sensitivity. In addition, object recognition procedures typically involve a working memory component and the identification of irregular shapes might require a visual reference implying that these identification tasks are not pure measures of haptic function (Newnham and McKenzie, 1993).

Researchers have used robotic manipulanda to examine haptic curvature perception (Sciutti et al., 2010; Gori et al., 2012) and other geometries (Henriques and Soechting, 2003, 2005). These robots are expensive, have a large footprint, require specialized personnel, and they are not portable. These are all factors that limit implementation of such systems in clinical settings. The haptic curvature assessment presented here fits in a rolling case for easy transportation and storage.

A pitfall of many psychophysical procedures is the large number of trials required to obtain a threshold. We employed a relatively new psychophysical threshold adaptive algorithm originally designed for visual perception research that required fewer trials to estimate a perceptual threshold which significantly improves the time requirements of the psychophysical haptic threshold assessment (Prins, 2013). Given that clinical personnel operate under narrow time constraints, any time-consuming assessment procedure (>15 min) is prohibitive and will likely not be implemented.

### Clinical Application of the Instrument

The lack of clinically appropriate, high-resolution measures of somatosensory function makes it difficult to perform consistent assessments over time to identify and monitor somatosensory function in neurologic populations (Moore and Groninger, 2013; Haryani et al., 2017; Mohrmann et al., 2017). The haptic assessment presented here has the potential for broad application in research and clinical settings as it meets the need for a quick, objective, high-resolution assessment of somatosensory or, more specifically, haptic function. The normative data on haptic sensitivity and acuity presented here provide a basis for identifying and quantifying haptic impairment in adult patient populations.

With respect to the clinical validity of the instrument, we here demonstrated its usefulness in characterizing haptic function in individuals with suspected CIPN. While each individual in this group had exposure to vinca alkaloids, the diagnosis, time since diagnosis, and treatments were unique for each individual. Despite this heterogeneity, the majority demonstrated elevated thresholds (67% above the 75th percentile of the normative cohort) and two of these individuals demonstrated haptic impairment (above the 95th percentile). This assessment also successfully quantified mild-to-moderate haptic impairment in a pediatric population with developmental coordination disorder compared to an age-matched cohort (Tseng et al., 2019). In summary, there is evidence indicating that the instrument can detect and differentiate between unaffected perception, and mild or more severe forms of haptic loss.

## Conclusion

The Minnesota Haptic Function Test™ generates valid measures of haptic sensitivity and acuity in agreement with previously reported curvature perception measures. There are numerous neurological populations with central and peripheral nervous system conditions that would benefit from the application of a widely accepted assessment to identify and monitor changes in haptic function. Here, we demonstrate that the assessment has sufficient resolution and test-retest reliability to (1) objectively quantify haptic function and (2) identify haptic impairment in both adult and pediatric neurological populations. In addition to having sufficient resolution, this haptic function measure is well suited for clinical application as the instructions are easy to understand, the assessments are quick to complete, the system has minimal space requirements and is easily portable.

## Ethics Statement

The study protocol was approved by and carried out in accordance with the requirements of the Internal Review Board of the University of Minnesota. Data collection occurred at the University of Minnesota campus and at the University of Minnesota Masonic Children’s Hospital. All participants gave written informed consent in accordance with the Declaration of Helsinki.

## Author Contributions

The manuscript was prepared by JH-W and edited by Y-TT and JK. Data collection was overseen by JH-W. The assessment method described here was designed by JK, Y-TT, and JH-W.

### Conflict of Interest Statement

The authors declare that the research was conducted in the absence of any commercial or financial relationships that could be construed as a potential conflict of interest.
